# Retraction Note to: Exercise-induced mitochondrial p53 repairs mtDNA mutations in mutator mice

**DOI:** 10.1186/s13395-021-00264-7

**Published:** 2021-03-30

**Authors:** Adeel Safdar, Konstantin Khrapko, James M. Flynn, Ayesha Saleem, Michael De Lisio, Adam P. W. Johnston, Yevgenya Kratysberg, Imtiaz A. Samjoo, Yu Kitaoka, Daniel I. Ogborn, Jonathan P. Little, Sandeep Raha, Gianni Parise, Mahmood Akhtar, Bart P. Hettinga, Glenn C. Rowe, Zoltan Arany, Tomas A. Prolla, Mark A. Tarnopolsky

**Affiliations:** 1 Department of Kinesiology, McMaster University, Hamilton, ON L8N 3Z5 Canada; 2 Department of Pediatrics, McMaster University, Hamilton, ON L8N 3Z5 Canada; 3 Department of Medicine, McMaster University, Hamilton, ON L8N 3Z5 Canada; 4 Northeastern University, Boston, MA 02115 USA; 5 Buck Institute for Research on Aging, Novato, CA 94945 USA; 6 Department of Medical Sciences, McMaster University, Hamilton, ON L8N 3Z5 Canada; 7 School of Health and Exercise Sciences, University of British Columbia Okanagan, Kelowna, BC V1V 1V7 Canada; 8 Department of Medical Physics & Applied Radiation Sciences, McMaster University, Hamilton, ON L8N 3Z5 Canada; 9 Division of Cardiovascular Disease, University of Alabama, Birmingham, AL 35294 USA; 10 Perelman School of Medicine, University of Pennsylvania, Philadelphia, PA 19104 USA; 11 Departments of Genetics, University of Wisconsin, Madison, WI 53706 USA; 12 Departments of Medical Genetics, University of Wisconsin, Madison, WI 53706 USA

**Retraction note to: Skeletal Muscle (2016) 6:7**

**https://doi.org/10.1186/s13395-016-0075-9**

The Editors-in-Chief have retracted this article. Following concerns raised by the corresponding author, an investigation by McMaster University confirmed concerns with Figs. 3 and 4 of this article, specifically:

- Figure 3b: the VDAC band appears to be identical with the Actin band of Fig. 3b of the original article
- Figure 4d overlaps with Fig. 4b of the original article

The Editors therefore no longer have confidence in the reliability of the data reported in the article.

Adeel Safdar, Konstantin Khrapko, Ayesha Saleem, Michael De Lisio, Adam P. W. Johnston, Imtiaz A. Samjoo, Yu Kitaoka, Daniel I. Ogborn, Jonathan P. Little, Sandeep Raha, Gianni Parise, Mahmood Akhtar, Bart P. Hettinga, Glenn C. Rowe, Zoltan Arany and Mark A. Tarnopolsky agree to this retraction. James M. Flynn, Yevgenya Kratysberg and Tomas A. Prolla have not responded to correspondence about this retraction.

